# Supplementary material for: ChiMSource improves the accuracy of studies on novel amino acid sequences by predicting alternative sources of mass spectrometry-derived peptides
Source: Comput Struct Biotechnol J. 2025 Aug 20;27:3704–9. doi: 10.1016/j.csbj.2025.08.023 (PMC12799943; doi:10.1016/j.csbj.2025.08.023)
Supplement: Supplementary file 1 — Supplementary material [file mmc1.pdf]

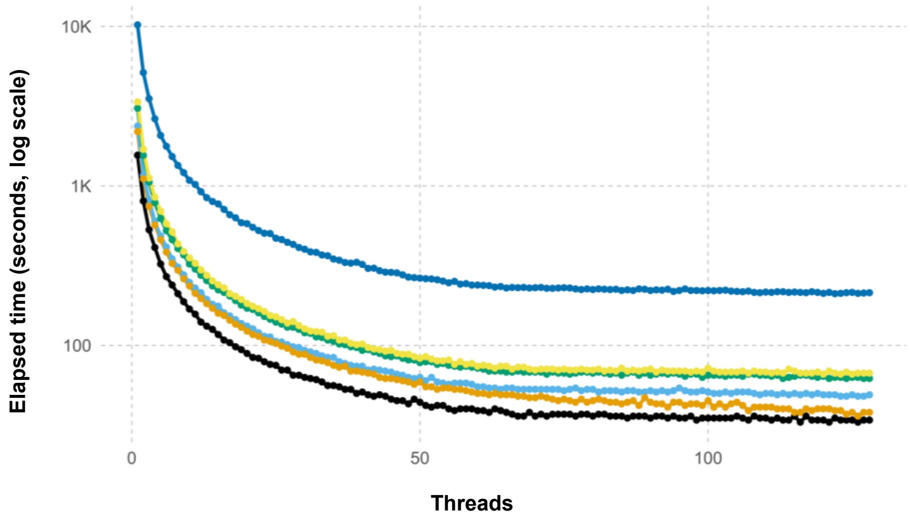

Legend:

Line color	Species	Number of transcripts	Transcriptome size, nt
	Homo sapiens	205,792	388,561,450
	Gallus gallus	44,937	152,771,019
	Danio rerio	57,775	121,902,979
	Arabidopsis thaliana	48,359	86,469,099
	Medicago truncatula	44,642	67,116,870
	Caenorhabditis elegans	34,689	56,317,021

Supplementary Figure S1. Runtime analysis of ChiMSource. The computational speed of ChiMSource is directly proportional to the transcriptome size and inversely proportional to the number of used threads. Transcriptomes were downloaded from Ensembl and EnsemblPlants.

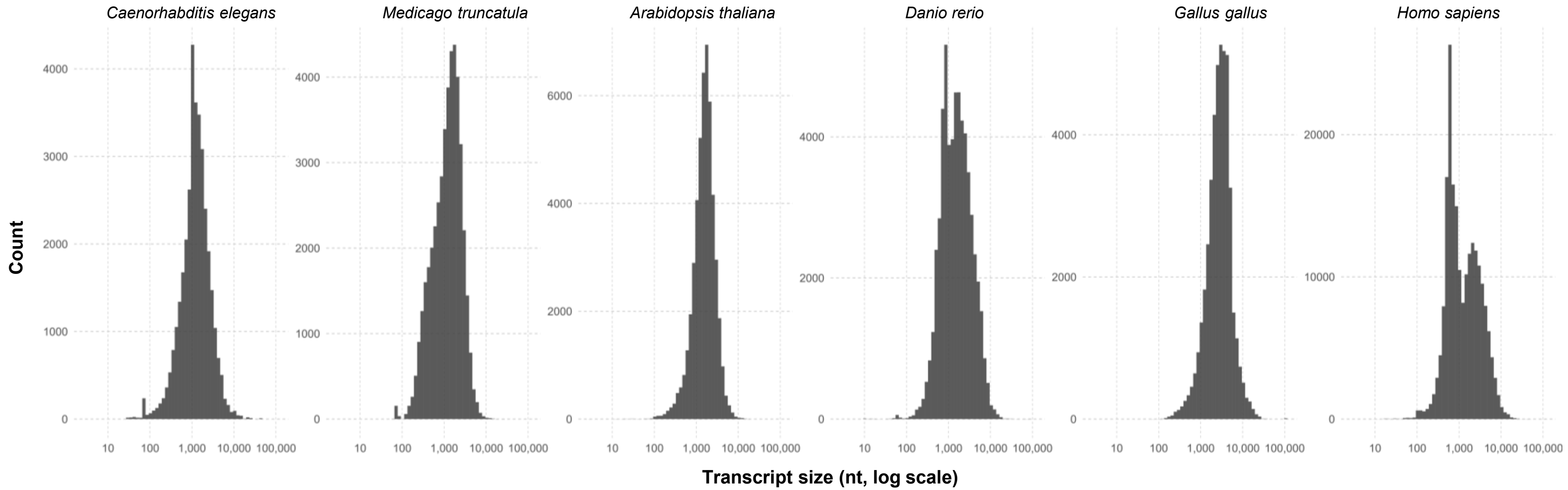

Supplementary Figure S2. Distribution of transcript lengths of six species used for benchmarking of ChiMSource. Note that transcriptomes available via Ensembl and EnsemblPlants contain some short non-mRNA transcripts along with mRNA transcripts (*C. elegans* and *M. truncatula*). Although short transcripts are processed quickly, the multithreading makes the process wait for the completion of longer transcripts, which results in a slower analysis of transcriptomes with many short transcripts included even if their total length in nucleotides is shorter compared to other genomes. This delay was not observed during the benchmarking of these six transcriptomes because the number of included short transcripts was small. However, it would be considerable if all non-mRNA transcripts were included (results not shown).
